# “*Even if she’s really sick at home*, *she will pretend that everything is fine*.”: Delays in seeking care and treatment for advanced HIV disease in Kinshasa, Democratic Republic of Congo

**DOI:** 10.1371/journal.pone.0211619

**Published:** 2019-02-13

**Authors:** Emilie Venables, Ilse Casteels, Elysée Manziasi Sumbi, Eric Goemaere

**Affiliations:** 1 *Médecins Sans Frontières*, Southern Africa Medical Unit (SAMU), Cape Town, South Africa; 2 Division of Social and Behavioural Sciences, School of Public Health and Family Medicine, University of Cape Town, Cape Town, South Africa; 3 *Médecins Sans Frontières*, Kinshasa, Democratic Republic of Congo; 4 Centre for Infectious Disease Epidemiology and Research, School of Public Health and Family Medicine, University of Cape Town, Cape Town, South Africa; Sefako Makgatho Health Sciences University, SOUTH AFRICA

## Abstract

**Introduction:**

HIV prevalence in the Democratic Republic of Congo (DRC) is estimated to be 1.2%, and access to HIV testing and treatment remains low across the country. Despite advances in treatment, HIV continues to be one of the main reasons for hospitalisation and death in low- and middle-income countries, including DRC, but the reasons why people delay seeking health-care when they are extremely sick remain little understood. People in Kinshasa, DRC, continue to present to health-care facilities in an advanced stage of HIV when they are close to death and needing intensive treatment.

**Methods:**

This qualitative study was conducted in one health-care facility in Kinshasa. A total of 24 in-depth interviews with purposively selected health-care workers, patients and care-givers were conducted. Patients were currently or previously hospitalised with advanced HIV, defined as CD4 count *<*200 cells/μl. Patients included those who had previously started antiretroviral treatment (ART), and those who had not. Participant observation was also carried out. Interviews were audio-recorded, translated from French and Lingala into English, transcribed, coded and thematically analysed using NVivo.

**Results:**

The main reasons for delaying access to health-care were stigmatisation, religious beliefs and limited economic resources. Stigmatisation meant that people feared disclosing their HIV status and thus did not receive support from their families. Religious leaders were reported to have encouraged people not to take ART. Patients delayed seeking treatment as they could not afford it, and health-care workers believed that staff at other facilities in Kinshasa were delaying HIV diagnoses for economic benefit.

**Conclusions:**

Delays in accessing care and treatment linked to stigma, religious beliefs and economic factors contribute to explaining the persistence of advanced HIV within this context. Access to free HIV-testing, ART and treatment of opportunistic infections; counselling; training of health-care workers; support for care-givers and stigma reduction strategies are urgently needed to prevent unnecessary deaths.

## Introduction

HIV prevalence in the Democratic Republic of Congo (DRC) is estimated to be 1.2%, which is lower than many other countries in Sub-Saharan Africa [1]. It is estimated that only one in two people living with HIV are diagnosed, and ART coverage (38%) is also among one the lowest in the region [2]. In Kinshasa, the capital city of DRC, HIV prevalence is estimated to be 1.6% of the population [1].

Advanced HIV disease is defined by a CD4 count <200 cells/μl or having stage 3/4 illness as classified by the WHO [3–5]. Patients with advanced HIV disease are often hospitalised in an extremely critical state, and hospitalisation may occur too late to prevent death. The enduring burden of advanced HIV disease has been recently described in this setting in DRC, as well as in Kenya and South Africa [5–8].

In a recent study conducted in the *Médecins Sans Frontières* (MSF)-run *Centre Hospitalier de Kabinda* (CHK) where this qualitative research took place, intra-hospital mortality from HIV was found to be extremely high. Of the patients hospitalised during 2014, 22.6% of them died, and of these, 68% were women. A third of these deaths (34%) occurred within 48 hours of admission. Of these patients, 78% had previously started ARV treatment, and their median amount of time on treatment was 74 months (six years) at the point of death. Factors linked with these deaths included treatment interruption of at least three months prior to hospitalisation; tuberculosis and a CD4 count <100 cells/μl [9]. Studies from other contexts have shown that almost 25% of patients interrupt their treatment at some point in their lives, and that these interruptions usually last between a few days and a few months [3]. Treatment interruption can lead to the patient developing drug resistance. A study conducted in Khayelitsha, South Africa, explored what the authors term ‘disengagement from care’, showing that 23 percent of ART patients disengaged from care at least once in a contemporary two year period but that 58 percent returned to care, or remained alive without hospitalisation [10].

Adherence challenges are often linked to treatment interruption. These challenges have been well-documented in other contexts, and have been linked to stigmatisation, disclosure, lack of financial means, difficulties in accessing health-care facilities, a lack of psycho-social support and complicated drug regimens [11–15]. In addition, there are specific challenges to adherence with children and adolescents such as lack of awareness of their HIV status, lack of knowledge about their treatment, lack of parental support and household conflicts [16].

A recently published systematic review of qualitative studies examined reasons why people living with HIV do not initiate treatment [17]. The review describes a range of factors including the health status of the individual, individual characteristics including their socio-demographic background, HIV-related stigma and social support, which was also linked to patients’ disclosure of their own HIV status [18–21]. Other factors affecting initiation included what the authors refer to as ‘supply-side factors’, which include long queues in facilities, drug stockouts and provider-patient interactions [20, 22].

This qualitative study, however, looks at the reasons why people delay seeking health-care services for advanced HIV, not only barriers affecting adherence, from the perspective of patients, health-care workers and care-givers. The study aimed to understand why people were arriving in CHK in such an advanced stage of the disease, with the intention of being able to use the findings to guide the care and support offered to patients and to prevent unnecessary deaths.

## Methods

### Setting and population

This study was carried out in one health-care facility, known as CHK, in Kinshasa. MSF has been providing HIV and TB care at CHK since 2002, including free in-patient and out-patient services. At the time of the study, the hospital had 38 beds for patients: 28 for general hospitalisation linked to HIV and another 10 for the isolation of TB patients. There is a general policy of paying for consultations, including consultations for HIV, at many health-care facilities in Kinshasa, but MSF provides services—including HIV consultations, treatment for opportunistic infections and for TB—for free.

### Sampling and recruitment

Purposive sampling was used to select patients (n = 7) and their care-givers (n = 8) from CHK. Some patients were hospitalised at the time of the interviews, whereas others were out-patients who had been previously hospitalised. Selected patients were discussed with the medical team of CHK before conducting interviews to ensure that the patient was not too sick to be interviewed. Care-givers stayed at the hospital, sleeping in a designated room, for prolonged periods of time to support their relatives. They were informed as a group about the study by one of the co-investigators who speaks Lingala and who was working as a psychologist in the project at the time of the study, and asked if they would like to participate. She knew some of the patients and care-givers through her daily work as a psychologist, but ensured that there was a separation between her duties providing counselling support and those as a researcher. Health-care workers (n = 9) were purposively selected by the research team, from a list of all staff, to ensure the inclusion of interviewees with a range of experiences and profiles including clinicians, nurses and counsellors. All potential participants were informed that the study was being conducted to learn more about delays in seeking care for advanced HIV, with the aim of improving future services and providing people with better support to prevent such delays. Potential participants were informed that both researchers worked with MSF, but that the PI did not work in the project and thus had no prior relationship with interviewees. All potential interviewees were approached face-to-face, apart from previously hospitalised patients who were contacted telephonically.

### Study design and procedures

This was an exploratory qualitative study using thematic analysis, which was carried out in October 2016. A total of 24 in-depth interviews were conducted with eight care-givers, seven patients and nine health-care workers. Participant observation was carried out by the Principal Investigator (PI) in the ward and in the area assigned for care-givers staying in the hospital in order to observe the daily running of CHK and the area in which care-givers stayed. All interviews were conducted in French or Lingala, in quiet, private areas within the hospital and its grounds. Interviews with health-care workers were conducted in French by the PI and interviews with care-givers and patients were conducted by the PI and a local Lingala-speaking co-investigator who translated into French during the interviews and participant observation. Interviews were conducted using in-depth interview guides which were developed with the research team and pre-tested before use to ensure accurate translation. Both the researchers involved in data collection were female and trained in qualitative research methodologies. The PI has a PhD and the co-investigator assisting with interviews, an MA. Interviews lasted between 9 and 59 minutes: some of the interviews were short as due to the extremely sick state of the patients and we were not able to reach saturation with the patient interviews as we did not want to over-burden them. In some cases, patients or care-givers were interviewed more than once if they consented, so as not to disturb their medical care or to enable them to rest. Care-givers, patients and health-care workers were all interviewed individually, except for one minor who requested the presence of their care-giver. No care-givers, patients or health-care workers refused to participate.

### Data analysis

All interviews were audio-recorded, translated into English and transcribed in a ‘one step’ process, where simultaneous translation and transcription occurred. Written notes were taken during the interviews and participant observation and these were referred to during the transcription and translation process to enrich the discussions and help the researchers build a complete picture of the context in which the interview had taken place. Results were discussed with the research team and CHK staff for verification: it was not possible to discuss the results with the patients or care-givers as they had all been discharged or were deceased at the time of analysis and could not be contacted further. All identifying data were removed during transcription. The demographic details of health-care workers have not been included to prevent them from being identified. Patients are identified only by their age and gender to protect their identities.

The transcripts were coded and analysed thematically by the PI, using NVivo qualitative data analysis software for this process. Data analysis was conducted by the PI and discussed with one of the other co-investigators. Thematic analysis was applied, in which the transcripts were read, reviewed and then organised into codes before interpretation took place. A total of 59 codes were developed during the analysis process: these codes were based on a thorough reading and re-reading of the transcripts, in which the PI looked for relevant information from the words of the interviewees. From these codes, main themes were extracted and the three main themes identified are presented below. Initial codes included adherence challenges; the role of care-givers in providing support; poverty; cost of treatment and perceptions of the responsibility for delaying seeking care: these were all merged into the three main themes of ‘stigmatisation’, ‘religious beliefs’ and ‘economic factors’. The data were continuously reflected upon by the PI and discussed with the other co-investigators during analysis in order to identify the key themes and any areas in which there was divergence.

The findings of this study have been reported following the Consolidated Criteria for Reporting Qualitative Research (COREQ) guidelines [23].

### Informed consent and ethical approval

Written informed consent/assent was taken from study participants before interviews began. In the case of the two minors aged under 18, written consent was taken from the parent/guardian and written assent from the minor. Interviews were carried out separately unless the minor requested the presence of their guardian during the interview. The study was approved by the MSF Ethical Review Board (1625) and the Ethical Review Board of the School of Public Health at the University of Kinshasa (ESP/CE/045/2016).

## Results

Of the eight care-givers who participated in the study, seven were female and one was male. Two clinicians, one community outreach staff member, two counsellors and four nurses were also interviewed. Seven patients were interviewed: six were female, and two were aged under 18.

As identified during the coding process, the three main reasons for delaying access to HIV care and treatment identified from interviews with patients, care-givers and health-care workers as well as from observations were stigmatisation, religious beliefs and economic factors.

### Stigmatisation

Patients, health-care workers and care-givers explained how the stigma surrounding HIV prevented people from seeking medical care and adhering to ART even when they were severely ill. HIV was still believed to be a synonym for death by many people in Kinshasa, as this health-care worker described:

Some people think that because they have HIV it’s the end and that they are going to die. They don’t want to do anything…especially when they arrive here really sick.

This 34 year old care-giver described how she felt when she learned that her teenage nephew was HIV positive:

It was a big shock… We were grieving, like he was dead. We cried, cried, cried. I asked why that had to happen to him. We’d already lost his mother. We’ve been attacked and we lost. It’s like we were keeping it to ourselves, but we knew that one day he would leave us. It was like he was dead. It was a big shock.

Stigma and the fear of being judged by others prevented people from disclosing their HIV status and seeking support from their family members and others close to them. Even within CHK where specialised services for HIV were provided, HIV was not always openly discussed between care-givers and patients, and care-givers did not always know the HIV status of the person they were caring for, if the patient had not disclosed to them. Care-givers felt powerless to help relatives who had not disclosed their HIV status and believed they could have prevented the severity of their condition if they had known the HIV status of their relative and been able to assist them earlier.

This 34 year old woman was caring for her mother, who died the week after the interview was conducted. She talked about why she thought her mother did not disclose her HIV status:

Ah! Maybe she was scared or embarrassed. Scared of us. Because before it was terrible to disclose that kind of news. You could even…if someone told you that you had HIV then you thought they could die immediately. Maybe she was thinking about that. For me, it’s just a sickness like any other. I thought that HIV was something normal. But for her, maybe she thought that telling the truth was…demoralising. If she’d have wanted to tell me the truth that would have made things move faster and would have stopped us being in this situation.

This 25 year old female patient reported that she stopped taking ART because she feared being stigmatised by her family:

When I got to Kinshasa I was really scared to tell my family that I was HIV positive. I didn’t know how they would react. I tried to keep it to myself, until they realised for themselves how sick I was. I was scared. I said it was pointless: why should I carry on living? I can’t work, I can’t have children, I can’t get married… I thought I was going to be like that until I died, so I thought it was better to stop taking the medication so that I could die. It would be better for everyone.

Counsellors and clinicians also stressed that ‘*self-stigmatisation’* was another reason people delayed seeking health-care when they were sick. Such delays then made it harder for them to provide the clinical support to patients that they wanted to give as they were only able to treat people who were already extremely sick.

### Religious beliefs

Religion and the role of charismatic churches was a strong theme in interviews with patients, care-givers and health-care workers. Religious beliefs and the influence of certain pastors caused patients to refuse or delay seeking appropriate medical care and to stop taking ART. Care-givers often felt powerless against these influences and felt unable to help relatives who did not want to seek medical advice. In one case, a woman described how her sister, who was hospitalised in CHK at the time of the study, would periodically ‘*disappear*’ to a church camp and interrupt her treatment.

Interviewees reported how local church leaders told people not to take ART as they could instead perform ‘*miracles*’ to cure them. Interviewees also described seeing powerful media campaigns in which people publicly testified to say that they have been cured of HIV without taking ART.

The care-giver stated that her sister stopped taking ART because of her religious beliefs:

She told me herself that the prophet told her not to take the treatment! He said that she couldn’t have HIV, that it wasn’t meant for her… She’s here [in CHK] because she wasn’t taking it. She stopped taking it. She stopped taking it. She said she wasn’t going to take it anymore because her prophet told her not to.

Similar stories were recounted by health-care workers:

There are others whose pastors tell them that they are cured. They stop their treatment immediately because of their beliefs. They tell them that they are cured, so there’s no reason to take the pills anymore. Sometimes they throw them away. Sometimes they just stop taking them.

This counsellor also described the detrimental impact that she believed religious beliefs had on patient adherence:

The biggest difficulty is churches…that has the biggest impact on adherence. The churches! They say not to take your medication anymore, that it’s a miracle, that you have been cured! The person leaves their treatment and then ends up here almost dead. Because of beliefs. False beliefs. We see lots of people who come here in an advanced state who refuse to be tested, who say it is witchcraft, who say they only need to pray to get better.

### Economic factors

Another important theme emerging from all categories of interviewees was how the cost of treatment and medication affected access to health-care services across Kinshasa. There was limited knowledge amongst patients and care-givers that MSF’s services were free, and a sense of regret at not knowing about them earlier:

I think it’s about money. I didn’t know before [that CHK is free]. I didn’t know the medication was free. If I had known that my mother had HIV, and if I had known that MSF gave drugs for free, I could have brought her here. I could have brought her here. It’s free. You don’t pay anything! It’s free. Maybe these other people here who waited, maybe they don’t have any money… Everywhere else you pay. You pay, you pay, you pay.

A mother who was caring for her hospitalised son explained the costs involved in treatment at other health-care facilities in Kinshasa:

[T]hey said to do this, to pay that… They told us to buy serum for the drip. We came home after paying everything. And despite all of that, he was still ill. We spent $80 but there was no difference in him.

Health-care workers in CHK believed that some staff at other health-care facilities purposefully avoided diagnosing people with HIV so that they could benefit financially. If the person was diagnosed with HIV without first undergoing multiple tests and consultations, interviewees argued that the clinician would make less money, therefore delaying a diagnosis was considered to be more profitable.

A counsellor working in CHK also believed that health-care workers in other facilities could make a profit from their patients:

The health-care facility is like a *boutique* [shop]. They realise that if they request an HIV test, it’s going to block them [from making money]. They try to find ways to make money out of people who are sick. One example is that they do tests for various things and they give you treatment. Maybe they treat your diarrhoea, typhoid, malaria but they don’t want to talk to you about HIV. They prefer to prescribe you medication which you pay for…they take the money.

These words were repeated by this nurse who linked the advanced stage of HIV in which patients arrived with the cost of health-care:

They have to pay for the treatment, for the examinations. They are asked for money and when they don’t have money, they arrive here in an advanced stage.

A male care-giver whose wife died during the course of the study explained that the decision to seek medical assistance depends on how sick the person is. If someone does not have the financial means to access health-care services, they wait until the condition is critical before seeking care:

Someone who is sick doesn’t know what’s happening to them. They don’t know what’s happening inside their body. All they feel is a fever. So he goes to the pharmacy, buys some tablets and swallows them. But there is something more serious going on, but they don’t have the means to seek proper treatment. But if the sickness gets worse, then you really need to do tests and that’s when you start to look for ways of taking someone to hospital.

## Discussion

HIV is still one of the main causes of hospitalisation and death in low- and middle-income countries, including DRC. Earlier presentation at health facilities enables clinicians to provide medical care and treatment, prevent unnecessary deaths and in turn can help prevent transmission to others, thus understanding the reasons for delaying seeking treatment is an essential part of service provision.

Our study provides a unique insight into people living with advanced HIV disease in Kinshasa and we discovered alarming reasonswhy people do not seek treatment earlier. Stigmatisation, religious beliefs and economic factors combined to create an environment where people were reluctant to seek medical care for themselves or their relatives and thus delayed doing so.

It is also important to consider that many of the patients in this study had cyclical relationships with care: health-care workers interviewed also expressed their sadness and frustration at seeing patients repeatedly hospitalised or having ongoing challenges with adherence to treatment. During one day of observation, a previously hospitalised patient was re-admitted, and staff welcomed her back whilst also being saddened to see her again. As Kaplan et al. describe in their study in Cape Town, patients who disengage from HIV care have an increased risk of poor health outcomes, but also increase the risk of transmitting HIV to others and developing drug resistance [10].

Stigma and discrimination have long-been common themes in HIV research, with HIV stigma often linked to a strong moral discourse around sexuality and sexual behaviour [24, 25]. Stigmatisation and stories of fear, ostracism and discrimination prevented people in Kinshasa from seeking HIV diagnoses, disclosing to those around them, starting and adhering to treatment and seeking medical assistance when they are ill, which then contribute to premature deaths. What is interesting to note in Kinshasa—and which should be addressed throughout the city—is that the stigma surrounding HIV is not only about delaying testing or not adhering to treatment, but is also related to seeking care for advanced disease. The general population, as well as those living with HIV, need to be made aware that advanced HIV does not have to be synonymous with death, and that there are facilities which are able to assist even when someone is severely ill.

Considering the implementation of innovative tools such as self-testing or peer-led HIV testing and support for people living with HIV may also reduce some of the stigmatisation surrounding testing by normalising it and making it more available to people who may otherwise be reluctant to test in a health-care facility [26]. In addition, providing people living with HIV with differentiated models of care such as clubs or Community ART Groups (CAGs) in which they can access ART outside of a health-care facility and receive peer support from others living with HIV is one way of reducing stigma [27–29]. Offering counselling and psycho-social support to people living with HIV can also help people manage the stigma they may face in their everyday lives: at present, counselling services for people living with HIV in Kinshasa are limited.

Religion plays a very prominent role for people living in Kinshasa, as elsewhere: it is an important support structure and source of hope, but at the same time, our data show how it can be damaging for those living with HIV. In this study, religion was, for many, clearly linked to a refusal to seek health care or take ART even when patients were extremely sick and close to death. Data presented above show the powerful influence of churches and the detrimental impact that certain religious beliefs, such as telling people that they have been ‘*cured*’ can have upon adherence to treatment. Other studies in settings including Malawi have, however, highlighted how religious leaders can have a positive influence on the behaviour of people living with HIV [25, 30–32]. Counsellors are also placed in a difficult position as they simultaneously respect the religious beliefs of each individual patient, whilst seeing the detrimental effect that religious beliefs can have in certain circumstances. We can also see how church leaders are in positions of power in their communities, with many of them having a very visual presence and large networks of followers. Informing and educating such leaders about HIV is one way of benefitting from their influence to encourage people to seek medical support earlier.

We cannot ignore the effect that cost has upon people’s ability—and willingness—to seek treatment for HIV earlier. As we presented above, the decision to seek medical assistance is influenced by the economic situation of the individual and their family: many of the patients who arrive in CHK were brought by a family member as they were too sick, in some cases already unconscious, to make the decision about seeking health-care services themselves. With HIV often being associated with death, spending money on medical assistance was described as a ‘*waste*’ if the individual was believed to be dying. Practically, across Kinshasa, it is important to advertise free HIV testing and treatment, so that people are not deterred from seeking assistance and are also able to ask for more information from their health-care providers when tests or treatment are offered to them. We also cannot ignore the reports of health-care workers using the health-care system to make money from patients, and who are believed to purposefully delay HIV diagnoses in order to gain financially.

This study has a number of limitations. Hospitalised patients were extremely sick and many eligible participants were too ill or distressed to be interviewed, meaning that the sample size was limited and saturation may not have been reached amongst patients. Due to the limited understanding of many of the patients’ diagnoses (by the patients themselves and their care-givers), it was not possible to know precisely when and where they accessed treatment or for how long. In addition, as all interviews took place in a hospital environment, interviewees may have been influenced by their surroundings and unwilling to give critical responses about their treatment or care. Despite these limitations, this study has many strengths, one of which being that we were able to explore delays in seeking treatment from the perspective of patients, their care-givers and the staff caring for them. Our qualitative data were very rich and we found strong thematic similarities across all groups of interviewee. This is one of the only studies the authors are aware of looking at people’s experiences of advanced HIV in Kinshasa and contributes to a growing body of literature looking at advanced HIV in low- and middle-income countries.

## Conclusions

The factors described above create an environment which makes it extremely difficult to live with HIV in Kinshasa, and which contributes to the numbers of people hospitalised with advanced HIV. Presenting at a health-care facility such as CHK often occurs too late to prevent death in people with advanced HIV, and four patients who participated in the study, or whose care-givers were interviewed, died during or shortly after the two-week period of data collection.

Preventing people from arriving at health-care facilities with advanced HIV requires a combination of wide-scale stigma reduction and information campaigns as well as an increased effort surrounding HIV testing and counselling, and treatment literacy. Economic barriers also need to be addressed to prevent HIV being profitable within the health-care system. Working with churches and using the powerful influence of local religious leaders could also help to encourage people to adhere to ART, seek treatment earlier and avoid unnecessary deaths. A more flexible understanding of individual patient journeys is required to reflect the experiences of the many patients who move in and out of care in their lifetimes [10] and to implement and offer services that reflect the cyclical relationship they have with ART. A focus on patient empowerment would also enable patients and their families to become more knowledgeable about HIV services and treatment and overcome stigma in order to ease their access into—or back into—the health-care system.
